# A New Mechanism of Dynamic Phase Transformations in An Isothermal Forged Beta–Gamma Intermetallic Alloy

**DOI:** 10.3390/ma12172787

**Published:** 2019-08-30

**Authors:** Zhengang Zhang, Shoujiang Qu, Guorong Cui, Aihan Feng, Jun Shen, Daolun Chen

**Affiliations:** 1 School of Materials Science and Engineering, Tongji University, Shanghai 201804, China; 2 School of Materials Science and Engineering, Harbin Institute of Technology, Weihai 264209, China; 3 College of Mechatronics and Control Engineering, Shenzhen University, Shenzhen 518060, China; 4 Department of Mechanical and Industrial Engineering, Ryerson University, Toronto, ON M5B 2K3, Canada

**Keywords:** titanium aluminide, phase transformation, precipitation, microstructure

## Abstract

A new mechanism of dynamic phase transformations of α_2_ ↔ γ in an isothermally forged γ-TiAl-based alloy that occur simultaneously during a short-term exposure at 1000 °C is identified in this study. In the heating process, γ phase significantly decreases through a phase transformation of γ → α_2_, while new γ lamellae are precipitated in the interior of equiaxed grains of α_2_ phase through a phase transformation of α_2_ → γ. The reasons for the presence of these two inverse phase transformations α_2_ ↔ γ occurring simultaneously are discussed.

## 1. Introduction

Intermetallic γ-TiAl-based lightweight alloys are well-suited for the aerospace applications since they combine a low density with high strength and superior oxidation resistance at elevated temperature [1,2,3]. β-solidifying TiAl-based alloys usually have a better superplastic ability than traditional two-phase (γ + α_2_) TiAl alloys, as the β/β_0_ phase (also called as B2 phase) can provide more slip systems at high temperature [4]. The temperature for the typical applications of TiAl-based alloys ranges from 600 °C to 900 °C [5], while the minimum temperature of phase transformation (labeled as T_min_) is ~1160 °C [6]. Huang et al. [7] reported that α_2_ lamellae in the as-HIP TiAl alloys decomposed through phase transformations of α_2_ → γ, α_2_ → B2 (or B2 + ω) and α_2_ + γ → B2 (or B2 + ω) when exposed at 700 °C for 10,000 h. Therefore, TiAl-based alloys without thermal deformation are metastable thermodynamically after annealing for a long time at a temperature below T_min_. In the process of forging or rolling at high temperature, stress-induced phase transformation [8], temperature-induced phase transformation, dynamic recovery and dynamic recrystallization occur simultaneously [9,10]. The fraction of γ, α_2_ and B2 phases varies with forging temperature and deformation amount of ingot [11]. To prevent grain growth, the wrought cakes are usually air-cooled to room temperature after forging, which do not have sufficient time to reach the phase equilibrium at low temperature. How the microstructure of these forgings changes during heating below T_min_ needs to be understood. This study is aimed at understanding the phase transformation mechanisms of isothermally forged beta–gamma TiAl-based alloy heated to 1000 °C and attempting to infer the stress-induced phase transformations in the forging process.

## 2. Materials and Methods

**Figure 1:**
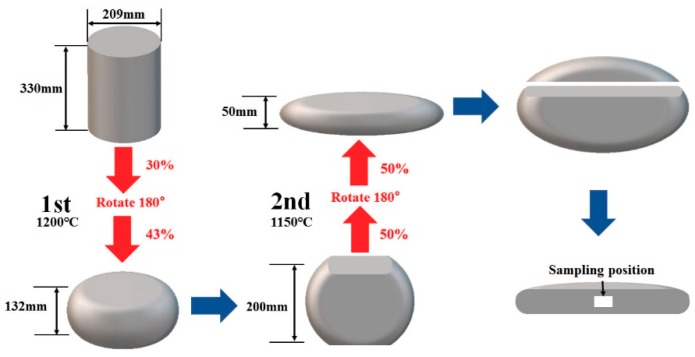
Schematic diagram of the two-step multi-directional isothermal forging and sampling position in the forged plate.

In the present study, the beta–gamma TiAl-based alloy with a nominal composition of Ti-44Al-4Nb-1.5Cr-0.1Mo-0.1B (at.%) was prepared by double vacuum consumable arc melting and then hot isostatically pressed (HIP) at 1300 °C under 180 MPa for 2 h followed by furnace cooling (FC). As shown in Figure 1, the cylindrical sample (Φ 209 mm × 330 mm) was preheated at 1200 °C for 6 h, and then immediately isothermal forged (ISF) at 1070 °C twice with a rotation of 180° before the second compression, with a total strain of 60% at a rate of 0.005 s^−1^, followed by air cooling (AC). Another 75% strain with the same process as the previous ISF was imposed on the forged cake after it was rotated 90° and preheated at 1150 °C for 6 h. Annealing was finally performed on the forged cake at 1000 °C for 2 h, followed by FC to 800 °C and AC to room temperature. Specimens of the isothermally forged plate from the white zone in Figure 1 were cut to a dimension of approximately 10 × 8 × 2 mm. The samples coated with high temperature paint (K-01 glass slurry) were heated at several selected temperature, followed by AC. Electro-polishing of specimens for light microscope (LM) and scanning electron microscope (SEM) observations was performed in a solution of 6% perchloric acid, 34% n-butyl alcohol, and 60% methanol electrolyte at 45 V and −30°C. The surface for LM observations was etched in a hydro-solution containing 3 vol.% HF and 5 vol.% HNO_3_. X-ray diffraction (XRD, Rigaku D/Max-2550) with Cu *K*_α_ radiation (λ = 1.5418 Å) was used to identify the phases at 50 kV and 200 mA with diffraction angles (2θ) from 10° to 100° with a step size of 0.02° and 1 s in each step. SEM (Nano SEM 450, FEI Company, Hillsboro, OR, USA), transmission electron microscopy (TEM; Tecnai G2 S-Twin F20, FEI Company, Hillsboro, OR, USA), high-resolution transmission electron microscopy (HRTEM), high-angle annular dark-field scanning transmission electron microscopy (HAADF-STEM; E.A. Fischione Instruments, Westmoreland, PA, USA) and energy-dispersive X-ray spectroscopy (EDS) were conducted on the samples to identify the mechanisms of phase transformation. Thin foils for TEM were prepared through mechanical polishing to a thickness of 100–120 μm and then twin-jet electro-polishing with a solution of 6% perchloric acid, 34% n-butyl alcohol, and 60% methanol electrolyte at 45 V and −30 °C. The hardness of samples was tested using a Vickers hardness tester (HVS-1000A; Huayin, Laizhou, Shandong, China) with a load of 4.9 N for 12 s.

## 3. Results and Discussion

### 3.1. Microstructure Analysis of Forged Samples before and after Heat Treatment (HT)

**Figure 2:**
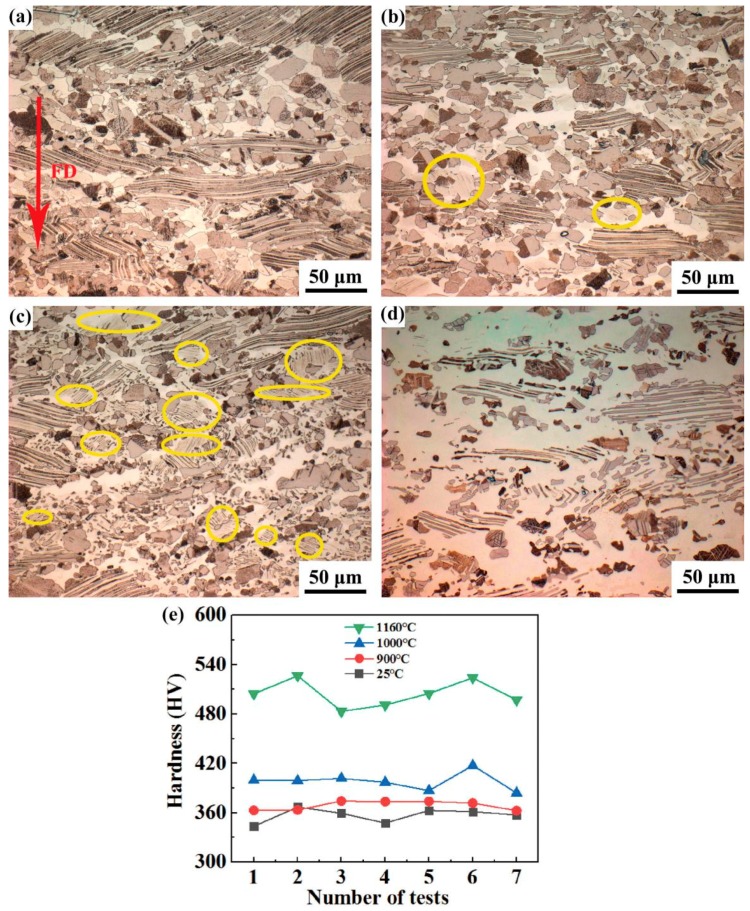
Microstructure of isothermally forged TiAl samples in different states: (**a**) forged sample; (**b**) heating at 900 °C for 6 h; (**c**) heating at 1000 °C for 1 h; (**d**) heating at 1160 °C for 1 h. The last-step isothermal forging direction (FD) is marked (**a**); (**e**) the hardness of the forged samples in various conditions corresponding to samples (**a**–**d**).

As seen from Figure 2a, the microstructure of the isothermally forged alloy consists of fine grains and γ/α_2_ lamellar colonies. The γ/α_2_ lamellar colonies were twisted or broken due to isothermal forging, and the remaining broken lamellae seemed to extend perpendicular to the last-step forging direction. Similar results were reported in [11]. After heating at 900 °C for 6 h, it can be seen from Figure 2b that the microstructure of the forged alloy has hardly changed, suggesting good thermal stability. Moreover, a new kind of acicular lamellae, which were a little blurry, appeared in some grains circled in yellow. Figure 2c shows the microstructure of sample after heating at 1000 °C for 1 h, where two obvious changes occurred: (i) A lot of smaller grains with a diameter of <5 μm appeared in the areas of initial equiaxed grains, and (ii) More acicular lamellae, circled in Figure 2c, were extensively presented in the alloy. The newly-precipitated lamellae (NPL) were thinner than the initial lamellae and were not twisted. However, they were not observed in the sample after heating at 1160 °C for 1h (Figure 2d), where the structure has vanished obviously.

Figure 2e shows the hardness values of the four samples, each of which has been tested seven times. On the whole, the hardness of the forged sample tended to increase with increasing heating temperature. Specifically, the hardness of the sample heated at 900 °C for 6 h was very close to that of the sample without experiencing heating, which was consistent with the microstructural observation above. As the microstructure changed from 1000 to 1160 °C, the hardness increased more obviously. It has been reported that the relationship among the hardness values of the three phases in the alloy was β_o_ > α_2_ > γ [12]. This suggests that the content of γ phase in the forged sample was more likely to be reduced if phase transformation occurred during the heat treatment and caused the hardness to increase. 

### 3.2. Phase Transformation Studied with XRD

**Figure 3:**
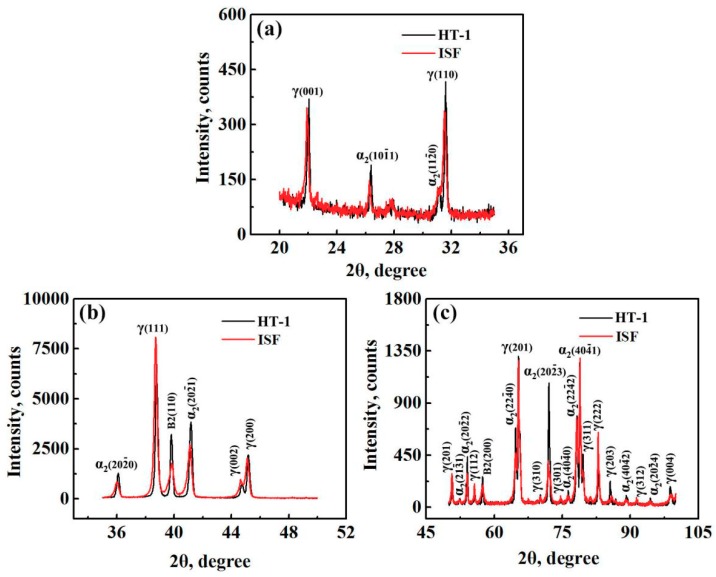
Comparison of X-ray diffraction (XRD) patterns between the samples in the states of isothermal forging (ISF) and isothermal forging + 1000 °C/1 h/AC (HT-1) (**a**) 20° < 2θ < 35°; (**b**) 35° < 2θ < 50°; (**c**) 50° < 2θ < 100°.

Since obvious microstructural change occurred in the sample heated at 1000 °C, which was close to the upper limit of the service temperature of TiAl alloy, the forged sample heated at 1000 °C for 1 h (HT-1) was selected to compare with the initial forged sample. Significant changes are observed via XRD after HT-1. As shown in Figure 3, after heating the intensity increased for γ(001), γ(110), γ(203) and γ(004), while decreased for γ(111), γ(201), γ(112) and γ(222) and changed little for others. Almost all the intensities of α_2_ phase increased except for α_2_(11$\bar{2}$0), which was separated from γ(110) with almost no change. As for the intensity of B2 phases, both B2(110) and B2(200) peaks increased after HT-1. Generally, XRD diffraction intensity will increase as the phase content increases. Therefore, the content of α_2_ phase and B2 phase increased after HT-1. Since only three kinds of phases can be identified in the alloy, it can be inferred that the total amount of γ phase decreased even if the intensity of some peaks increased, which is consistent with the three highest peaks of α_2_, γ and B2 lying in-between 35–45°, suggesting that the refinement of grains in Figure 2c is actually a result of the dissolution of γ grains through γ → α_2_ + B2. 

The rate of phase transformation will be very slow when the temperature is below T_min_ [13]. Since the forged cake was heated at 1150 °C for 6 h before the final-step forging (which lasted almost 160 s), and then was directly air-cooled after forging at 1070 °C, there was no sufficient time for the atomic diffusion to reach equilibrium considering its large size. In theory, only if the effect of temperature on the phase transition is considered, γ lamellae should have been precipitated from the α_2_ phase [14] and increased during heating at 1000 °C through $\alpha_{2}^{ss}$ → α_2_ + γ according to the phase diagram [4]. This was actually confirmed since it happened in the small pieces in the heating process in the later part of this study. Then, the occurrence of γ → α_2_ + B2 phase transition during heat treatment at 1000 °C is an abnormal phenomenon. During forging at high temperature, stress-induced phase transformation, mainly including γ → α_2_, α_2_ → γ and α_2_ → B2, will occur in the TiAl-based alloy [8,15,16]. In the (α + γ + β/B2) phase field, different strain amounts lead to different fractions of phases, and the fraction of γ phase increases with increasing strain at the same temperature [11,17]. In other words, the atomic diffusion caused by stress-induced phase transformation in a short time is faster than that caused by temperature, and α_2_ → γ transformation may be easier and faster than γ → α_2_. However, there is no direct evidence to prove this up to now.

### 3.3. Analysis of Newly Precipitated γ Lamellae

**Figure 4:**
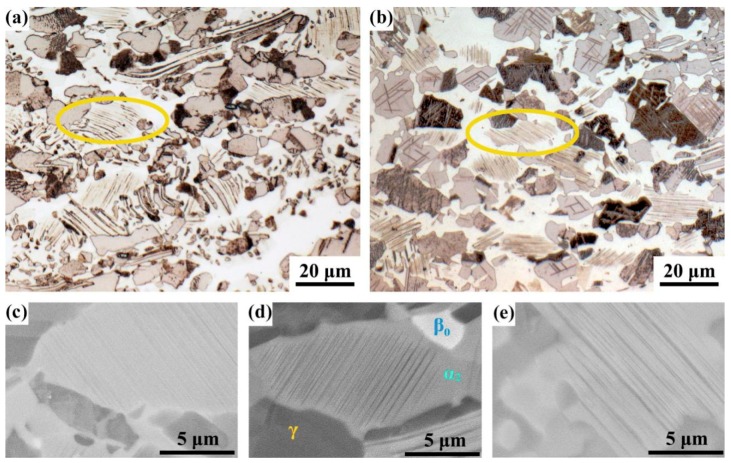
Light microscope (LM) images (**a**,**b**) and BSE images (**c**–**e**) of newly precipitated lamellae in the forged samples of HT-1 (**a**,**d**), HT-24 (**b**,**e**) and sample with no heating process (**c**).

To study the precipitation mechanism of lamellae at 1000 °C, a forged sample heated at 1000 °C for 24 h (HT-24) was used to compare with the sample HT-1. As circled in Figure 4a,b, the NPL became denser and clearer after heating at 1000 °C for 24 h. Figure 4c,d and e show the BSE (backscattered electron) images of α_2_ grains in the initial forged sample and the samples of HT-1 and HT-24, where the dark phase is γ phase, the gray one is α_2_ phase, and the white one is B2 phase. The NPL were not obvious in the initial forged TiAl (Figure 4c) and coarsened at heating process (Figure 4d,e). In a word, it is beneficial to keep the forged sample heated at 1000 °C for the precipitation of lamellae.

**Figure 5:**
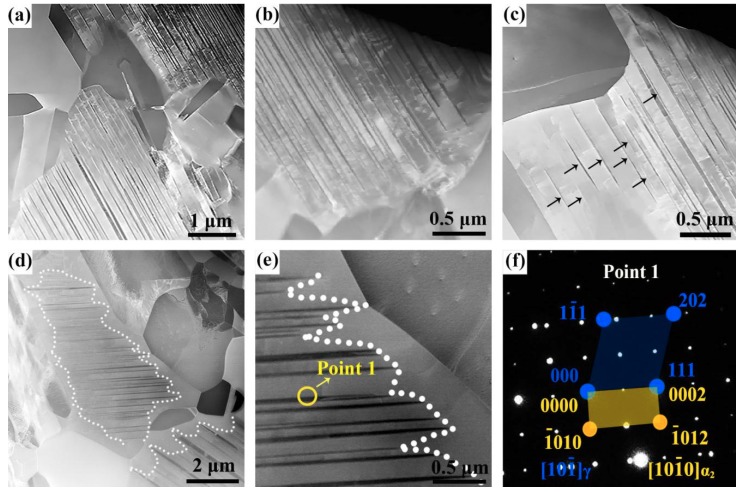
The high-angle annular dark-field (HAADF) images of initial isothermally forged TiAl (**a**–**c**) and HT-1 (**d**,**e**), where (**f**) is the selected area diffraction pattern from location 1 in (**e**).

Figure 5a–c shows the high-angle annular dark-field (HAADF) image of NPL in α_2_ grains observed in the initial forged sample. The precipitated lamellae are parallel to each other and are less than 100 nm in thickness, which cannot be observed under LM. The nano-thickness NPL in α_2_ grains may be formed by the previous annealing after forging. Most of the NPL almost reach the grain boundary (Figure 5a), but it can be seen that the NPL near the grain boundary are finer and somewhat needle-like. Some NPL cannot reach the grain boundary of α_2_ grains, and the density in the interior of α_2_ grain is larger than that near the grain boundary (Figure 5b). The coarsest part of NPL is inside the grain and both ends of some NPL did not reach the grain boundary, as indicated by the arrows in Figure 5c. It follows that NPL first appeared inside the α_2_ grains rather than the grain boundary, which is different from the mechanism reported in [18]. After heating at 1000 °C for 1 h, the grains of α_2_ phase grew up by devouring the γ grains, resulting in both ends of NPL being farther away from the grain boundary, which can be seen in Figure 5d. Interestingly, the NPL inside the grain were not swallowed by the α_2_ phase but thicker than those before the heat treatment (Figure 5e). This means that NPL were continuously precipitated inside the α_2_ grains while α_2_ grains devoured γ grains, that is, α_2_ → γ and γ → α_2_ phase transformations occurred simultaneously or dynamically in the process of heat treatment. Figure 5f shows a selected area electron diffraction (SAED) pattern of position 1 in Figure 5e under TEM, revealing that the NPL and the parent phase maintain the Blackburn relationship: (111)γ//$\left(0001\right)\alpha_{2}$.

### 3.4. Atomic Diffusion in HT-1 Sample

EDX analysis under HADDF was performed in the samples before and after heat treatment, with at least three points in each phase determined. As seen from Table 1, after heat treatment the concentration of Al increased in α_2_ phase, and decreased in equiaxed γ phase. The concentration difference may provide a driving force for γ → α_2_ in the heating process at 1000 °C. However, the atomic percent of NPL inside α_2_ grains increased slightly, which is different from the equiaxed γ grains. This means that the NPL have nothing to do with the initial equiaxed γ grains and are not precipitated from grain boundaries. Thus, the increase of Al concentration in α_2_ phase should be the only reason to promote the precipitation and coarsening of γ lamellae (Figure 5e), except that the stacking fault itself can easily cause the segregation of solute elements. The higher Al concentration in the equiaxed γ grains of the initial forged sample may be attributed to a stress-induced phase transition, which needs further study.

### 3.5. Mechanisms of Dynamic Phase Transformation

**Figure 6:**
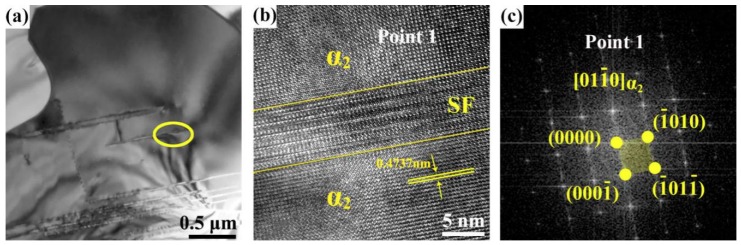
(**a**) Transmission electron microscopy (TEM) image of precipitated lamellae in the interior of α_2_ grain, (**b**) high-resolution transmission electron microscopy (HRTEM) image of stacking faults, (**c**) Fast Fourier Transformation (FFT) pattern of point 1 in (**b**).

Figure 6a shows the TEM image of NPL in the sample after heat treatment. To figure out the precipitation mechanism of γ lamellae, the portion of a relatively thin and short precipitated lamella circled in Figure 6a was observed via HRTEM. As shown in Figure 6b, this lamella is not γ phase but a stacking fault of α_2_ phase. Figure 6c shows the Fast Fourier Transformation (FFT) image of α_2_ phase (point 1 in Figure 6b). It has been reported that the nucleation of γ-lamellae proceeds from grain boundaries or stacking faults on the (0001)α_2_ basal plane [14]. There are lots of different points of view on the formation mechanism of stacking faults in α_2_ phase [19,20,21,22], where the main view is that stacking faults originate from grain boundaries or are caused by the cross-slip of Shockley dislocations from grain boundaries to basal planes [23]. Due to the order-disorder phase transition of α_2_ → α, the *c/a* ratio of α_2_-phase decreases when the temperature approaches or exceeds T_min_ (being 1160 °C) [24], which may be another reason for the formation of stacking faults in α_2_ phase. Once the internal stacking faults of α_2_ phase are formed, the element segregation would occur near them at a high temperature. This is also the reason why lamellae can be precipitated when the concentration of Al in α_2_ phase is low (Table 1). The newly grown areas of α_2_ grains in the heating process at 1000 °C (Figure 5d,e) do not experience the process of forging or order-disorder phase transition, thus there are no conditions for stacking faults to form in these freshly grown areas. Besides, the newly formed α_2_ phase is close to the equilibrium state at 1000 °C, so the element supersaturation is absent there. It follows that NPL do not continue to extend after heat treatment, but only coarsen after reaching the original grain boundary, as shown by the dotted curves in Figure 5d,e.

**Figure 7:**
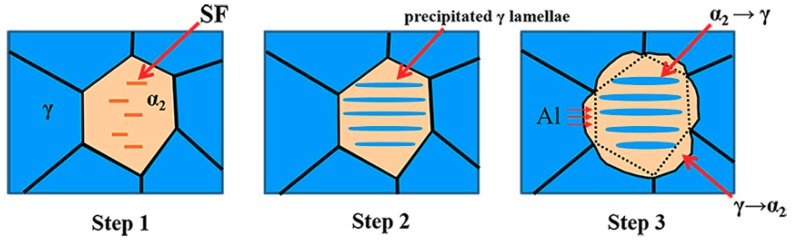
Schematic illustration of phase transitions α_2_ ↔ γ proposed in this study.

Figure 7 shows the whole process of phase transformations of α_2_ ↔ γ observed in this study. First, a large number of stacking faults occur inside α_2_ grains in the process of forging or order-disorder phase transition of α → α_2_. Second, Al atoms in the α_2_ grains are preferred to migrate into the stacking faults, forming the nano-thickness lamellae under the action of temperature after forging. Third, when heated to 1000 °C, Al atoms in γ grains migrate into α_2_ grains, causing γ grains to be dissolved and gradually transformed into α_2_ phase, and the Al atoms entering the α_2_ phase will further gather at the stacking faults of α_2_ phase and newly formed γ lamellae, which promotes the precipitation and coarsening of the γ lamellae in the α_2_ grains. 

## 4. Conclusions

The microstructure of the forged beta–gamma intermetallic alloy changed obviously because of phase transformation when heating in the range of 1000–1160 °C, leading to the increase in the hardness with increasing heating temperature. The stress-induced phase transformation in the forging process decreased the concentration of Al in α_2_ grains and increased the volume fraction of γ phase in a short time, providing a driving force for the dissolution of γ-phase in the forged TiAl-based alloy through γ → α_2_ + B2 phase transformation when heated at 1000 °C, which was a result of decomposition of Al in γ grains. The precipitation of γ lamellae in the α_2_ grains through α_2_ → γ was due to the preferential enrichment of Al at stacking faults. In a word, the dynamic phase transformations of α_2_ → γ and γ → α_2_ occur simultaneously.

## Figures and Tables

**Table 1:** Atomic percent (at.%) of Ti and Al elements in α_2_ phase and γ phase in the isothermally forged TiAl alloy and the isothermally forged sample after heat treatment at 1000 °C for 1 h.

State	α_2_ Phase		γ Phase			
			Equiaxed Grains		Precipitated Lamellae	
	Ti	Al	Ti	Al	Ti	Al
ISF	64.3	35.7	47.7	52.3	49.1	50.9
ISF + 1000 °C/1 h/AC	60.2	39.8	48.9	51.1	48.2	51.8

## References

[1] Appel F., Paul J., Oehring M. (2011). Gamma Titanium Aluminides: Science and Technology.

[2] Wu G.D., Cui G.R., Qu S.J., Feng A.H., Cao G.J., Ge B.H., Xiang H.P., Shen J., Chen D.L. (2019). High-temperature oxidation mechanisms of nano-/submicro-scale lamellar structures in an intermetallic alloy. Scripta Mater..

[3] Qu S.J., Tang S.Q., Feng A.H., Feng C., Shen J., Chen D.L. (2018). Microstructural evolution and high-temperature oxidation mechanisms of a titanium aluminide based alloy. Acta Mater..

[4] Qiu C., Liu Y., Zhang W., Liu B., Liang X. (2012). Development of a Nb-free TiAl-based intermetallics with a low-temperature superplasticity. Intermetallics.

[5] Huang Z.W. (2013). Thermal stability of Ti-44Al-4Nb-4Hf-0.2Si-1B alloy. Intermetallics.

[6] Schwaighofer E., Clemens H., Mayer S., Lindemann J., Klose J., Smarsly W., Güther V. (2014). Microstructural design and mechanical properties of a cast and heattreated intermetallic multi-phase γ-TiAl-based alloy. Intermetallics.

[7] Huang Z.W., Lin J.P., Sun H.L. (2017). Microstructural changes and mechanical behaviour of a near lamellar γ-TiAl alloy during long-term exposure at 700 °C. Intermetallics.

[8] Derder C., Bonnet R., Pe´nisson J.M., Frommeyer G. (1997). Evidence of stress-induced α_2_ → γ transformation in a Ti-30 at.%Al alloy. Scripta Mater..

[9] Zhang W.J., Lorenz U., Appel F. (2000). Recovery, recrystallization and phase transformations during thermomechanical processing and treatment of TiAl-based alloys. Acta Mater..

[10] Zhu K., Qu S.J., Feng A.H., Sun J.L., Shen J. (2019). Microstructural evolution and refinement mechanism of a beta–gamma TiAl-based alloy during multidirectional isothermal forging. Materials.

[11] Zhu K., Qu S.J., Feng A.H., Sun J.L., Shen J. (2018). Evolution of the microstructure and lamellar orientation of a β-Solidifying γ-TiAl-based alloy during hot compression. Metals.

[12] Schloffer M., Iqbal F., Gabrisch H., Schwaighofer E., Schimansky F.P., Mayer S., Stark A., Lippmann T., Göken M., Pyczak F. (2012). Microstructure development and hardness of a powder metallurgical multi phase γ-TiAl-based alloy. Intermetallics.

[13] Erdely P., Werner R., Schwaighofer E., Clemens H., Mayer S. (2015). In-situ study of the time-temperature-transformation behaviour of a multi-phase intermetallic β-stabilised TiAl alloy. Intermetallics.

[14] Wei D.X., Koizumi Y., Nagasako M., Chiba A. (2017). Refinement of lamellar structures in Ti-Al alloy. Acta Mater..

[15] Du X.W., Zhu J., Zhang X., Cheng Z.Y., Kim Y.W. (2000). Creep induced α_2_ → B2 phase transformation in a fully-lamellar TiAl alloy. Scripta Mater..

[16] Wang J.G., Chen G.L., Zhang L.C., Ye H.Q. (1997). Study on the stress-induced γ + α_2_ transformation in a hot-deformed Ti-45Al-10Nb alloy by high-resolution transmission electron microscopy. Mater. Lett..

[17] Liu S.Q., Shen J. (2018). Microstructural evolution and high-temperature oxidation mechanisms of a titanium aluminide based alloy. Mater. Res. Express.

[18] Qin G.W., Hao S., Sun X.D. (1998). Ledge mechanism of primary α_2_/γ lamellae growing in the supersaturated α_2_ matrix for γ-TiAl-based (γ +α_2_) alloy. Scripta Mater..

[19] Zghal S., Thomas M., Naka S., Finel A., Couret A. (2005). Phase transformations in TiAl-based alloys. Acta Mater..

[20] Denquin A., Naka S. (1996). Phase transformation mechanisms involved in two-phase TiAl-based alloys-Ⅱ. discontinuous coarsening and massive-type transformation. Acta Mater..

[21] Denquin A., Naka S. (1996). Phase transformation mechanisms involved in two-phase TiAl-based alloys-Ⅰ, lamellar structure formation. Acta Mater..

[22] Karadge M., Gouma P., Philos I. (2006). A structural aspect of α(α_2_) → lamellar α_2_ + γ transformation in γ-TiAl. Mag. Lett..

[23] Koizumi Y., Fujita T., Minamino Y., Hata S. (2010). Effects of plastic deformation on lamellar structure formation in Ti-39 at.% Al single crystals. Acta Mater..

[24] Yeoh L.A., Liss K.D., Bartels A., Chladil H., Avdeev M., Clemens H., Gerling R., Buslaps T. (2007). In situ high-energy X-ray diffraction study and quantitative phase analysis in the α + γ phase field of titanium aluminides. Scripta Mater..

